# Supplementary material for: The Origin and Maintenance of Tuberculosis Is Explained by the Induction of Smear-Negative Disease in the Paleolithic
Source: Pathogens. 2022 Mar 17;11(3):366. doi: 10.3390/pathogens11030366 (PMC8955375; doi:10.3390/pathogens11030366)
Supplement: Supplementary file 1 [file pathogens-11-00366-s001.zip › pathogens-1616375-supplementary.pdf]

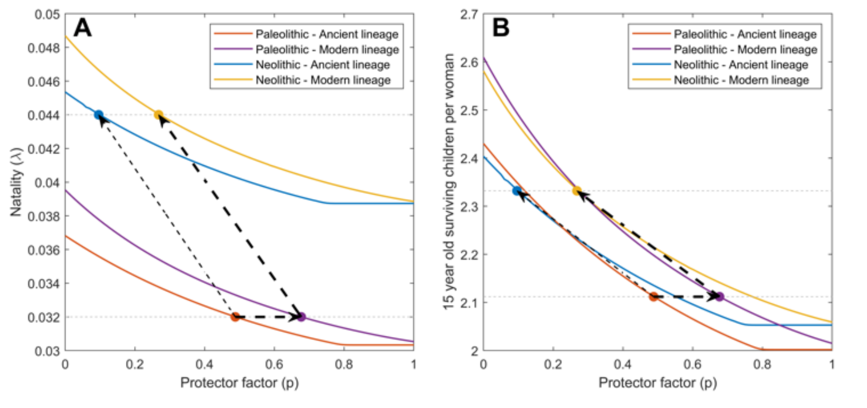

Figure S1. Hypothetical evolution of the Mtb lineages in relation to the protective factor and natality. Adjustment of natality (**A**) and effective natality understood as the number of births surviving more than 15 years (**B**) using the parameters of the TBSpectr model and the recon growth population for the Paleolithic (0.0003%/year) and the Neolithic (0.1%/year). Colored lines represent the values for the Paleolithic period and the infections with ancient (orange) and modern (violet) lineages of Mtb, as well as the Neolithic period and the infections with ancient (blue) and modern (yellow) lineages of Mtb. Dots of each corresponding color show the value of the protection factor (p) chosen in each case.

Arrows show the evolution of Mtb lineages with the protector factor. Thicker arrows show the mainstream evolution from the ancient towards the modern lineages favored by its decreased virulence, increasing the host protector factor, which was maintained when the Neolithic revolution brought about poorer health conditions.

The thinner arrows show the pathway of ancient lineages, impacting only those people with worse health conditions (lower p), thereby being less competitive.
